# Highlights from the 59th Bürgenstock Conference on Stereochemistry 2026

**DOI:** 10.1039/d6sc90190e

**Published:** 2026-10-19

**Authors:** Giacomo E. M. Crisenza, Guanqi Qiu

**Affiliations:** a Department of Chemistry, The University of Manchester Manchester M13 9PL UK giacomo.crisenza@manchester.ac.uk; b Max-Planck-Institut für Kohlenforschung Kaiser-Wilhelm-Platz 1 45470 Mülheim an der Ruhr Germany gqiu@kofo.mpg.de

## Abstract

Herein, we share an overview of the scientific highlights from speakers at the latest edition of the longstanding Bürgenstock Conference.
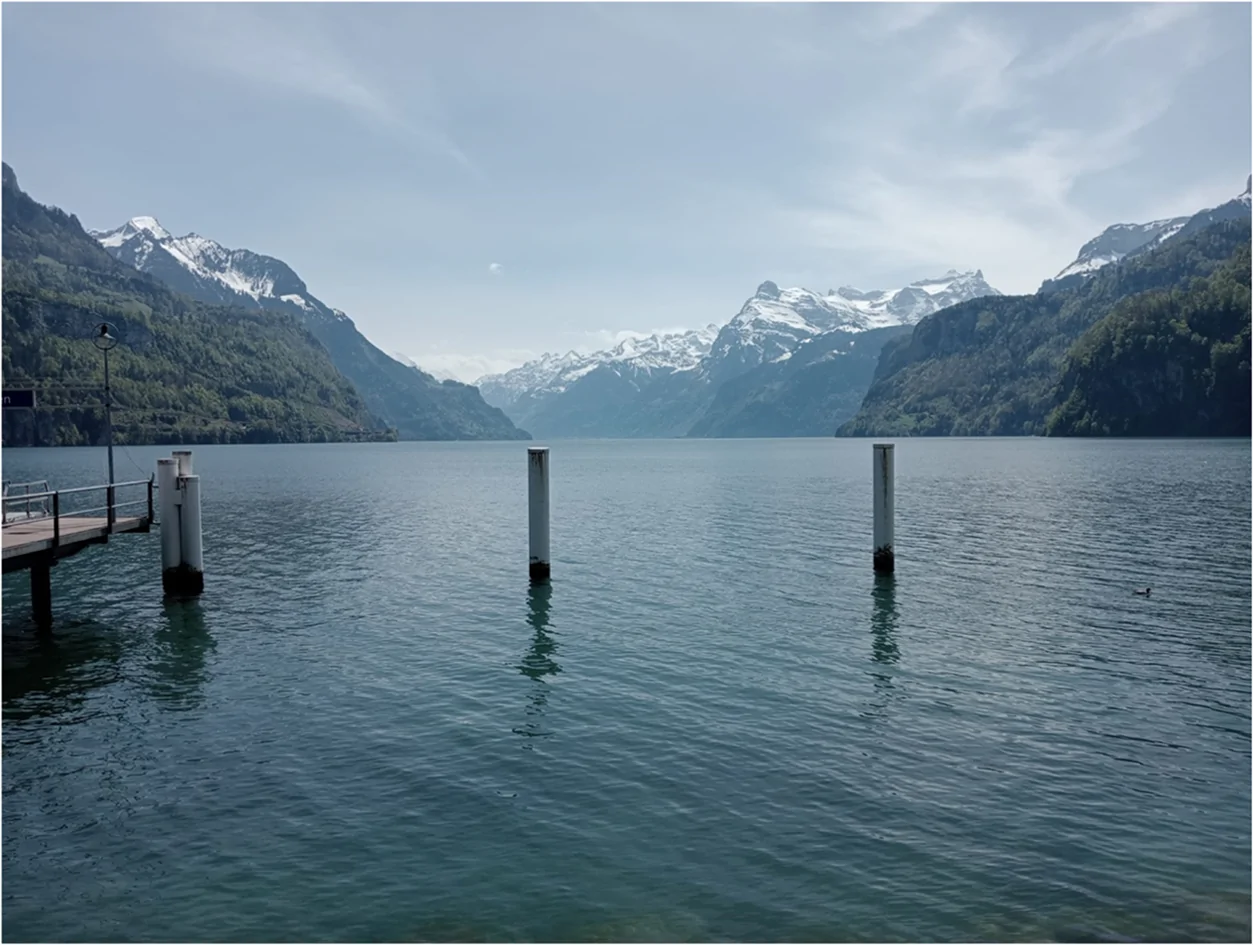

“*The following biography is intended to deal not only with the external course of my professional life; it also proposes to reveal the eroticism of scientific research: discovering – inventing – understanding.*” (Albert Eschenmoser in *Lives in Chemistry*, “*Prizes are Good but Discoveries are Better*”; copy donated to all 2026 JSP Fellows).

## Introduction

As every year, the Swiss springtime heralds the start of the Bürgenstock Conference, a flagship meeting in synthesis and catalysis in Europe and beyond, marking its 59th edition. The conference brings together scientists from across Europe and around the world to share the latest discoveries in cutting-edge areas of chemical research, extending far beyond its traditional roots in stereochemistry and asymmetric synthesis. This broad scientific vision was also reflected in this year’s edition, led by Prof. Paolo Melchiorre – conference President – whose programme spanned a broad range of disciplines, from organic synthesis and catalysis to chemical biology and materials science.

The conference experience begins well before the official registration – and the suspenseful release of the attendees’ list. In the hours leading up to the meeting, attendees gather in the charming village of Brunnen on the shores of Lake Lucerne, reconnecting with old friends, meeting new colleagues, and setting the stage for the stimulating discussions to come. Following registration, Prof. Jérôme Waser, coordinator of the Bürgenstock Conference JSP Programme, welcomed the JSP awardees from Europe, America and Asia to a dedicated networking get-together event. The gathering provided an opportunity for these young researchers to meet one another, as well as the conference President and Organising Committee. The JSP programme plays a vital role in promoting outstanding early-career scientists and has, over the years, supported generations of emerging researchers, many of whom later returned to the Bürgenstock Conference as invited speakers – a testament to the programme’s exceptional quality and impact.

After the JSP reception, the Fellows joined the rest of the participants for the inaugural dinner, highlighted by the traditional “balcony speech” delivered by Prof. Melchiorre. In his opening remarks, he officially welcomed attendees and announced the start of the conference. He also introduced the Vice-President of this edition, Prof. Varinder Aggarwal, who will serve as President of the next Bürgenstock Conference, as well as the Guest of Honour, Prof. Karl Anker Jørgensen, and greeted the members of the Organising Committee for facilitating the conference organisation. Prof. Melchiorre concluded by encouraging all participants to engage in thoughtful discussion and open exchange of ideas throughout the week, reaffirming the collaborative, friendly and intellectually adventurous spirit that has long defined the Bürgenstock Conference and that he sought to preserve in this year’s edition.

## The Cu-rious chemistry of common functionalities

Following the inaugural dinner, attendees gathered in the Mythen-Saal for the opening scientific session, chaired by Prof. Varinder Aggarwal, who introduced Prof. Tehshik Yoon (University of Wisconsin–Madison) as the first speaker of the conference. In a lecture entitled “*Uncommon Reactions of Common Functional Groups*”, Prof. Yoon presented his group’s efforts to unlock new radical reactivity from ubiquitous polar functional groups – including alcohols, amines and carboxylic acids – commonly found in pharmaceuticals and bioactive molecules.

Prof. Yoon’s research program leverages the rich photochemistry of simple coordination complexes that self-assemble from these compounds and inexpensive first-row transition-metal salts.^1,2^ One facet of the work focuses on the oxidative coupling of carboxylic acids, where a Cu(ii) or Fe(iii) salt facilitates the photochemical decarboxylative generation of organic radical intermediates and mediates their oxidative coupling with nucleophilic partners.^3^ Another thrust of the talk leveraged the high reactivity of photogenerated heteroatom-centred radicals to trigger radical fragmentation pathways, providing a new platform for the oxidative functionalisation of carbon–carbon bonds *via* formal heteroatom insertion into carbocyclic frameworks. This strategy offers streamlined access to pharmaceutically relevant heterocycles from readily available carbocyclic precursors.^4^

The talk offered a compelling reminder that photochemistry is not simply a new activation mode but a powerful way to reimagine – and invent – new reactions by harnessing the untapped synthetic potential of common functional groups traditionally regarded as chemically inert (including even the C–C bond!), thereby continuing to expand the synthetic toolbox available to organic chemists.

## A RAFT of solutions for polymer deconstruction

The Monday morning session, chaired by Prof. Jovana Milić, opened with the lecture by Prof. Athina Anastasaki (ETH Zurich) on innovative strategies for chemical recycling under the theme “*Monomers from Polymers*”. After introducing the pressing challenges associated with plastic waste disposal and recycling, and the limitations of current recycling strategies – mostly dominated by “downcycling” pyrolysis and mechanical recycling – Prof. Anastasaki highlighted chemical recycling as an emerging and underexploited technology capable of precise polymer degradation and of recovering monomers from end-of-life plastics, thus enabling a truly circular economy for materials.

To address this challenge, the Anastasaki group has developed controlled radical depolymerisation strategies based on reversible addition-fragmentation chain-transfer (RAFT)^5^ manifolds to achieve high control over polymer structure and composition during deconstruction. By pre-installing RAFT-active functionalities – such as xanthates – into polymer backbones, radical addition-fragmentation processes can be initiated, enabling the controlled release of monomer units over time.^6^ These methods enable the selective deconstruction of polyacrylates, including complex copolymers and sequence-defined multiblock materials. The group further demonstrated that Brønsted acid catalysis significantly enhances RAFT polymerisation efficiency, lowering initiator requirements and enabling the synthesis of well-defined multiblock copolymers with diverse acrylate and acrylamide compositions.^7^

Despite these advances, these depolymerisation strategies relied on bespoke polymers containing pre-installed end-group functionalities and were therefore not readily applicable to commercial materials. To this end, Prof. Anastasaki then described a photochemical depolymerisation strategy applicable to commercial methacrylate-based plastics. The method employs photogenerated chlorine radicals to abstract hydrogen atoms from the main polymer backbone, thereby triggering controlled depolymerisation and enabling the clean recovery of acrylate monomers, even from materials containing additives and dyes.^8^ Following initial studies exploiting photolysis of chlorinated solvents, more sustainable variants based on FeCl_3_ photoactivation were developed, while ongoing work is exploring alternative organic hydrogen-atom-transfer (HAT) reagents to further expand the scope and sustainability of these polymer recycling technologies.

Overall, Prof. Anastasaki’s lecture showed how precise control over radical reactivity can turn polymerisation chemistry on its head, transforming methods originally designed to build polymers into powerful tools for their controlled deconstruction.

## Nature’s blueprint: a breath of sunshine for artificial photosynthesis

Following the coffee break, the morning session continued with Prof. Marcella Bonchio (University of Padova), who presented her group’s research on the design of supramolecular hybrid materials mimicking the hierarchical architecture of natural photosynthetic membranes^9^ in a lecture entitled “*Supramolecular Hybrid Materials for Artificial Photosynthesis*”.

The lecture first introduced the concept of “quantasomes” as artificial analogues of Photosystem II, drawing inspiration from the structural and functional organisation of natural thylakoid membranes, and allowing the conversion of light quanta into chemical energy.^10^ To this end, she showcased the design of multi-chromophore networks built from perylenes bisimides, and combined with tetra-ruthenate oxygenic clusters. This hybrid material is shaped by supramolecular assembly, with red-shifted light-harvesting efficiency, favourable exciton accumulation, and dynamic aggregation into 2D-paracrystalline domains.^10^ Prof. Bonchio further discussed how PEG functionalisation regulates water harvesting at the quantasome core, mimicking the role of water channels in the PSII protein environment, and how cross-linked PBI reticulation enables the formation of photoresponsive frameworks with enhanced oxygenic photosynthesis performance.^11,12^ Concluding her presentation, Prof. Bonchio reported on the electrocatalytic potential of polyoxometalate/carbon nanohorn interfaces for selective oxidation reactions, highlighting new frontiers for inorganic multi-redox clusters beyond water splitting.^13,14^

Overall, the lecture illustrated how supramolecular chemistry can bridge molecular design and functional materials, bringing artificial photosynthesis ever closer to emulating the remarkable efficiency and complexity of Nature’s energy-conversion machinery.

## (Photo)chemical reactivity in a nutshell

Following lunch, the afternoon session was opened by Prof. Rafal Klajn (Institute of Science and Technology Austria, ISTA), introduced by Prof. Sami Lakhdar. In a lecture entitled “*Chirality Propagation Across Length Scales*”, Prof. Klajn showcased his group’s recent advances in exploiting molecular confinement to control reactivity and selectivity.

The lecture began by presenting light-responsive colloidal nanoparticles that reversibly self-assemble into confined “nanoflasks” under UV irradiation and disassemble upon exposure to visible light.^15^ The size and chirality of these supramolecular containers can be tuned by designing tailored ligands on the constituent nanoparticles, thereby allowing guest molecules to be selectively and reversibly encapsulated. Within these confined environments, molecular reactivity can be profoundly altered, leading to accelerated reaction rates and stereochemical outcomes distinct from those observed in bulk solution.

Prof. Klajn then turned to structurally defined, water-soluble Pd-based molecular cages assembled from Pd complexes and triimidazolyl-phenyl ligands. These precisely engineered hosts enable selective control over photo-responsive molecules. A particularly elegant example involved the photoisomerisation of azobenzene photo-switches: while the cage accommodates two *trans*-azobenzene molecules, irradiation induces *trans*-to-*cis* isomerisation, forcing one of the bulkier *cis*-isomers to be expelled from the cavity.^16^ The same confinement strategy was subsequently extended to assemble homo-^17^ and hetero-dimeric^18^ aggregates of BODIPY dyes and anthracene quenchers, providing a versatile platform for modulating fluorescence through guest exchange and photoexcitation.

Building on these studies, the Klajn group developed a strategy termed *disequilibration by sensitisation under confinement* (DESC).^19^ By co-encapsulating a BODIPY photosensitizer and a *trans*-azobenzene photo-switch within the same Pd-cage, visible-light excitation of the BODIPY chromophore triggers energy transfer to the azobenzene, promoting selective isomerisation to the metastable *cis*-isomer. Steric constraints within the cage then expel the *cis*-product, driving the reaction away from equilibrium. Remarkably, the process can also be powered by sunlight, eliminating the need for high-energy UV irradiation.

The lecture concluded with the presentation of adaptive “Janus-like” Pd-cages capable of reversibly switching between tube- and capsule-shaped cavities in response to the geometry of the encapsulated guest (linear *trans*-azobenzene *vs.* tetrahedral boron–ate complexes).^20^ Prof. Klajn left the audience with a thought-provoking question: can these dynamic nanoflasks and molecular cages be regarded as “minimalist” synthetic enzymes, capable of controlling reactivity and selectivity through confinement in much the same way as Nature’s catalysts do?

## Monday’s short presentations, flash talks and poster session

The second afternoon session, chaired by Prof. Jérôme Waser, hosted the first round of short presentations by early-career researchers. The session saw Dr Antony Bigot showcase the use of light and enzymes as green and selective reagents for chemical transformations; Prof. Hugh Nakamura present electrochemical strategies for accessing non-canonical cyclic peptides; and Dr Mattia Silvi highlight innovative approaches to carbon-chain homologation through the design of new alkenyl radical acceptors.

The spotlight then shifted to the JSP fellows, who delivered a series of rapid-fire flash talks, including Prof. Midori Akiyama, Prof. Lena Barra, Dr Gregor Cremosnik, Dr Giacomo Crisenza, Prof. Munielle Delley, Prof. Nina Hartrampf, Dr Jola Pospech, Prof. Martin Tomanik and Prof. Johannes Walker, providing a glimpse of the breadth of science represented by this year’s fellowship programme. The momentum carried seamlessly into the first poster session, where discussions spanned from fluorinated cubanes, bridged polycycles and rigid hydrocarbon linkers to selective hydrogenations, cooperative tandem catalysis and complex heterocycle functionalisation. Other contributions highlighted enzyme and natural product discovery, encoded-library technologies, chemical protein synthesis and nucleobase-specific supramolecular recognition, alongside new reactivities in photocatalysis, electrochemistry, mechanochemistry and flow-based synthesis. Materials- and application-driven topics, including boron-based dynamic macromolecular architectures, hybrid perovskite frameworks, organelle-specific dyes and therapeutic discovery, further broadened the scope of the session. Overall, the posters showcased both fundamental reaction development and emerging applications. Animated discussions around the posters continued well beyond the allotted session, setting the tone for the collaborative and highly interactive spirit that would characterise the remainder of the conference.

## Deciphering a comet’s chemical inventory

After dinner, the Monday evening session transported the audience into outer space, requiring 3D glasses for an immersive journey to the nucleus of comet 67P/Churyumov–Gerasimenko. Introduced by Prof. Jérôme Lacour, Prof. Uwe Meierhenrich (Université Côte d’Azur) delivered a captivating lecture entitled “*The Origin of Biomolecular Asymmetry and ESA’s Cometary Rosetta Mission*”, recounting his collaboration with the European Space Agency to uncover the molecular origins of life.^21^

Drawing inspiration from the Rosetta Stone, the *Rosetta* mission sought to determine the organic chemical composition of comets and their implications for prebiotic chemistry. Prof. Meierhenrich’s contribution centred on developing analytical instrumentation to detect organic compounds on the comet’s icy nucleus and in its dust and plasma tail. The comet’s nucleus represents a peculiar, yet “ideal”, natural reactor – exposing molecules to cryogenic temperatures and energetic cosmic radiation that cannot be reproduced under conventional laboratory conditions. To recreate this environment on Earth, Prof. Meierhenrich and co-workers developed artificial cometary ice experiments, in which simple mixtures of water, carbon dioxide, carbon monoxide, methanol and ammonia were frozen under ultra-high vacuum and irradiated with ultraviolet light. These studies demonstrated the spontaneous formation of 16 amino acids,^22^ together with biologically relevant carbohydrates including ribose (key subunit of nucleic acids),^23^ supporting the hypothesis that essential prebiotic building blocks could have been delivered to the early Earth by comets and meteorites. He further discussed how circularly polarised light in space may have contributed to the emergence of biomolecular homochirality.^24^

The lecture then turned to the analytical instrumentation aboard ESA’s *Philae* lander module. Following Rosetta’s historic arrival at comet 67P in 2014 and the first soft landing on a comet nucleus (despite the lander ultimately coming to rest almost upside down), multidimensional gas chromatography analyses revealed an unexpectedly rich inventory of organic compounds, including several nitrogen-containing molecules and a few species – including methyl isocyanate, acetone, propionaldehyde and acetamide – never before detected in a comet.^25^ These findings provided an unprecedented glimpse into the molecular composition of the early Solar System and further strengthened the link between cometary chemistry and the origins of life on Earth.

Concluding a truly breathtaking presentation, enriched by spectacular 3D-images of the comet and its surface, Prof. Meierhenrich reflected on the collaborative effort behind the Rosetta mission with a message that resonated well beyond astrochemistry: “*Science needs to be international and cross-generational.*”

## Lighting up uranium’s HAT-trick

The Tuesday morning session, chaired by Dr Carla Obradors, opened with a lecture by Prof. Polly Arnold (UC Berkeley) entitled “*f-Block Photocatalysts for Inert Carbon-Element Bond Transformations*”. The talk highlighted the emerging potential of uranyl photocatalysts for the selective functionalisation of unactivated C–H bonds, demonstrating how the photochemistry of f-block elements can unlock new opportunities in hydrocarbon activation and manipulation.

Prof. Arnold first introduced how the uranyl ion, [UO_2_]^2+^, the most common form of uranium in the environment and conventionally regarded as inert towards oxo group chemistry, can be photoexcited by visible light into a long-lived excited state that is strongly oxidising.^26^ While even the simplest ion is able to cleave certain unactivated hydrocarbon C–H bonds, the development of this reactivity into controlled and catalytic C–H bond functionalization is still in its infancy. Prof. Arnold presented hydrocarbon-soluble, air-stable, tunable uranyl catalysts that are protected against unwanted excited-state quenching.^27^ Prof. Arnold showed the first substrate scope for uranyl photocatalysis, explained how ligand design can extend photoexcited lifetimes and reactivity,^28^ and can deliver new regioselective functionalization of unactivated hydrocarbons, for the first time.^29^ Prof. Arnold finished by showing a new simple dinuclear uranyl complex that, upon photoexcitation, can catalyse alkane-to-alkene transformations by hydrogen atom abstraction, a feature not possible with known mononuclear uranyl complexes.^30^

This lecture demonstrated that f-block catalysis has far more to offer than traditionally appreciated. Through the combination of bespoke ligand design and visible-light activation, uranyl complexes emerge as a new class of photocatalysts capable of unlocking the reactivity of otherwise unactivated molecules, opening exciting new avenues for selective C–H bond functionalisation.

## Seeing pnictogen chemistry in a new light

Following the coffee break, the Tuesday morning session continued with Prof. Sami Lakhdar (CNRS/Université Toulouse 3 Paul Sabatier), who presented his team’s recent investigations into the light-driven reactivity of pnictogen-based compounds and intermediates. In a lecture entitled “*Making and Breaking Chemical Bonds with Visible Light*”, Prof. Lakhdar guided the audience through three distinct topics: the reductive activation of *N*-alkoxypyridinium salts, the unconventional reactivity of strained phosphiranes, and the photoactivation of pincer-ligated P(v) complexes.

The first part of the lecture focused on *N*-alkoxypyridinium salts, employed either as electron acceptors in photoactive electron donor–acceptor (EDA) complexes or reduced *via* single-electron transfer from an excited-state photocatalyst to generate *O*-centred radicals. These intermediates served as versatile HAT agents to access P-, C- and Si-centred radicals, enabling alkyne-triggered radical cascades towards decorated benzo[*b*]phospholes^31^ or coupling with the pyridine fragmentation products to deliver alkylated and silylated *N*-heteroarenes.^32^ Alternatively, direct trapping of the alkoxy radicals with phosphites afforded phosphate esters,^33^ further highlighting the synthetic versatility of *N*-alkoxypyridinium salts.

Prof. Lakhdar then turned to the unusual reactivity of strained three-membered phosphiranes. Their polar reactivity is governed by preferential nucleophilic attack at either phosphorus or carbon, depending on the nucleophile’s nature – *N*-nucleophiles attacking at *P* and *O*-nucleophiles at C.^34^ Photochemical activation, however, offers opportunities to reshape these intrinsic reactivity patterns: addition of photogenerated carbenes to phosphiranes forms phosphorus ylides that redirect *O*-nucleophilic attack, promoting ring opening at carbon.^35^ Unpublished studies further indicated that direct photoexcitation of phosphiranes can generate highly reactive phosphinidenes (*i.e.* the phosphorus analogues of nitrenes), while interception of photoredox-generated N-centred radicals provides access to medicinally relevant phosphonamide derivatives.

In the final part of his lecture, Prof. Lakhdar explored whether P(iii)/P(v) redox cycles could mimic the elementary steps of transition-metal-catalysed cross-couplings. Using *O*,*N*,*O*-pincer-ligated phosphorus compounds, the group exploited light to both access high-valent P(v) intermediates and promote otherwise challenging ligand-coupling pathways. Photogenerated carbenes derived from acylsilanes undergo P–O bond insertion into the *O*,*N*,*O*-pincer-ligated P(iii)-complex, generating six-membered nucleophilic intermediates.^36^ These species intercept electrophilic partners (*e.g.* tropylium tetrafluoroborate) and rearrange to form photoactive acyl phosphoranes. Irradiation of the latter complexes releases acyl radicals, which undergo C–C bond-forming additions to alkenes while regenerating the original P(iii) species, thereby highlighting P-mediated couplings – and ultimately phosphorus catalysis – as a promising alternative to traditional transition-metal-catalysed processes.

Throughout Prof. Lakhdar’s lecture, detailed mechanistic investigations – encompassing computational studies, NMR and EPR spectroscopy and emission decay kinetics; among others – underscored a central message: a deep understanding of reaction mechanisms can not only rationalise reactivity, but also drive reaction discovery and development.

## Libiamo, libiamo ne’ lieti calici

Tuesday’s free-time afternoon offered a welcome opportunity to enjoy the spectacular surroundings of Lake Lucerne, with participants hiking, exploring Brunnen, playing sports or simply relaxing in the sunshine. After dinner, attention returned to the Mythen-Saal, where a piano had appeared on stage and glasses of Malvasia wine were flowing – setting the perfect mood for the forthcoming *Salon Evening*, hosted by Conference President Prof. Melchiorre.

The evening’s guest – Italian composer, pianist, musicologist, educator and music populariser Giovanni Bietti – took the Bürgenstock audience on a musical celebration of the *brindisi*: the art of raising a glass together. Combining engaging monologues with live piano performances, Bietti guided the audience on a journey across Europe and through centuries of music, from Haydn, Beethoven and Rossini to Verdi, Schumann, Čajkovskij, Puccini, Debussy and Bartók. Each composition offered its own tribute to wine, conviviality and the simple pleasure of sharing good times in good company. Among the memorable “take-home messages” offered by the evening were: “*Friends, water makes you dumb; we learn this from the fishes. But in the case of wine, the opposite is true*” (Haydn); or, for those preferring a less classical perspective, “*Friends and love can disappoint you; wine never will*” (popular saying). Sipping Malvasia, specially selected for the occasion, while enjoying this musical journey gave the evening a particularly immersive flavour and perfectly captured the spirit of the conference: a celebration of gathering together, exchanging thoughts and sharing good times over a glass of wine – or whatever beverage one might prefer!

## When silicon takes charge

The first Wednesday morning session, chaired by Prof. Luca Dell’Amico, opened with a lecture by Prof. Martin Oestreich (Technische Universität Berlin) entitled “*The Cation Shuffle*”. The talk presented a unifying catalytic concept in which proton, silylium, carbenium and arenium ion catalysis are interconverted, enabling challenging bond-forming and bond-breaking transformations through the controlled manipulation of highly reactive cationic intermediates.

Prof. Oestreich first discussed the Brønsted acid-mediated generation, isolation and characterisation^37,38^ of counteranion-stabilised silylium ions and the role of ligand scrambling in controlling the speciation and reactivity of these highly electrophilic silicon Lewis acids.^39,40^ The focus then moved to recent applications of this cationic interplay, including arenium-ion-catalysed halo-dealkylation of fully alkylated silanes, where formally inert tetra-organosilanes can be converted into functionalized silanes through a top-down strategy.^41^ Prof. Oestreich further highlighted how shuttling between proton and silylium-ion catalysis can address long-standing challenges in electrophilic aromatic substitution, including Friedel–Crafts C(sp^2^)–H methylation of arenes with methanol and chemoselective chloroalkylation processes.^42^ Finally, Prof. Oestreich showed how super-electrophile catalysis enables exhaustive H/D exchange of deactivated aryl halides under mild conditions, providing access to otherwise expensive or unavailable deuterated aryl halides for use as NMR solvents.^43^

Together, these examples illustrate that when carefully orchestrated, highly reactive cationic intermediates can be transformed from fleeting, seemingly unstable species into productive catalytic platforms.

## Complex building blocks made simple

The second Wednesday morning talk brought an industrial perspective to the session, with Dr Pavel Mykhailiuk (Enamine Ltd, “*the largest building block company on Earth*”) presenting his team’s efforts to expand the chemical space available for drug and agrochemical development. In a lecture entitled “*Saturated Heterocycles for Medicinal Chemistry*”, Dr Mykhailiuk showcased Enamine’s strategy of developing user-friendly, simple and scalable syntheses of complex building blocks, providing medicinal chemists with novel heterocyclic scaffolds and saturated bioisosteres for drug discovery.

The first part of the lecture focused on the design of practical reagents for the divergent synthesis of decorated N-heterocycles bearing medicinally relevant functionalities at positions that remain challenging to access using conventional strategies. Here, Dr Mykhailiuk highlighted the development of bis-electrophilic vinamidinium^44^ and enamino-aldehyde^45^ reagents, enabling straightforward access to methylsulfonyl- and fluorine-containing aza-heteroarenes from a broad range of bis-nucleophilic partners, rapidly expanding the diversity of compounds available for screening libraries. Complementary studies introduced bis-nucleophilic *O*-vinylhydroxylamine reagents,^46^ which engage aza-arene *N*-oxides in sequential *N*-arylation/[3,3]-sigmatropic rearrangement cascades to provide otherwise difficult-to-access azaindole derivatives. The same strategy was subsequently extended to alkynes to synthesise substituted pyrroles.^47^

The second half of the lecture centred on the discovery and scalable synthesis of strained saturated hetero- and carbocyclic scaffolds as bioisosteres for substituted aromatic rings and common nitrogen heterocycles. Dr Mykhailiuk presented a series of elegant synthetic solutions to access spirocyclic and bicyclic frameworks – including 1-azaspiro[3.3]heptanes,^48^ azabicyclo-hexanes, -heptanes and -octanes,^49^ bicyclo[1.1.1]pentanes,^50^ oxabicyclo[2.1.1]hexanes^51^ and spiro[3.3]heptanes^52^ – using diverse synthetic tools ranging from thermal and photochemical [2 + 2] cycloadditions, iodine-mediated intramolecular cyclisations, photochemical atom-transfer radical addition (ATRA) reactions, scalable flow photochemical processes, and ketene chemistry. Remarkably, these building blocks were used to prepare “patent-free” analogues of bioactive compounds, which were subsequently evaluated in biological assays. Many displayed improved metabolic stability and physicochemical properties while retaining biological activity, highlighting their value as saturated bioisosteres for medicinal chemistry.

Throughout the lecture, Dr Mykhailiuk emphasised the importance of balancing synthetic innovation with industrial practicality, acknowledging the gap that often exists between attractive lead structures and compounds that can be feasibly and, especially, economically manufactured at scale. This philosophy of “*simple synthetic strategies for complex building blocks*” underpins Enamine’s approach to reagent and building-block development. Looking ahead, he identified enantioselective synthesis as an important future direction, while noting that access to all stereoisomers remains highly valuable during early-stage drug discovery. Overall, the lecture demonstrated how the full synthetic toolbox can be harnessed to address challenges at the interface of academic methodology and industrial medicinal chemistry.

## Beyond Flatland: taking hydrocarbons into a new stereochemical dimension

Under the chairmanship of Prof. Tibor Soòs, the afternoon session opened with a lecture by Prof. Christof Sparr (University of Basel) entitled “*Stereoselective Formation and Opening of Aromatic Rings*”. The talk showcased the Sparr group’s efforts to challenge the conventional perception of aromatic rings as static, planar structural motifs by developing catalytic strategies for their stereoselective construction, selective cleavage and controlled three-dimensional functionalisation.

The lecture opened by illustrating the counterintuitive concept that stereoselective arene formation enables the preparation of an exceptional range of chiral aromatic structures *via* aldolizations, Wittig reactions, and metathesis.^53,54^ Prof. Spaar then showed that aromatic ring-opening metathesis (ArROM) enables the cleavage of aromatic rings and provides an efficient catalytic approach to transform and interconvert various aromatics.^55^ Another topic presented in the lecture focused on catalyst control over stereogenic units that encode more than two configurations.^56–60^ Besides the significantly larger stereochemical space that can be addressed by stereoselective synthesis in the realm of higher-order stereogenicity, the conceptual interconnection with conformational space was established. After presenting catalyst-stereocontrolled stereoselective syntheses of atropisomers, these concepts were translated to other stereogenic units.^61^ The lecture concluded with the stereoselective synthesis of *skew*-tetramantane,^62^ the chiral core of diamond, achieved through an ingenious adamantalogous “cage-extension” strategy that delivered the first controlled synthesis of this higher diamondoid.

The lecture demonstrated that aromaticity and caged carbon frameworks should no longer be regarded as static structural motifs, but rather as versatile platforms for stereoselective reactions. By learning to construct, manipulate and even selectively cleave aromatic rings and condensed carbocyclic cages, the Sparr group is redefining the role of hydrocarbons as dynamic three-dimensional scaffolds in modern asymmetric synthesis.

## Wednesday’s short presentations, flash talks and poster session

After the coffee break, the audience reconvened in the Mythen-Saal for Wednesday’s round of short presentations. Chaired by Prof. Jérôme Waser, the session featured Dr Durga Prasad Hari, introducing propellanes as unconventional carbene surrogates; Prof. Hong Guen Lee, presenting a convergent synthesis of the alkaloid (−)-phalarine; Dr Constanze Neumann, discussing how active-site size and metal organisation can be controlled in MOF catalysts; and Dr Christoph Schotes, highlighting Bayer’s industrial adoption of Pfaltz-type catalysts for enantioselective hydrogenation.

The short presentations were followed by rapid-fire flash talks from JSP fellows Prof. Mate Bezdek, Dr Elodie Brun, Prof. Xiaoqiang Huang, Prof. C. Rose Kennedy, Dr Daniele Mazzarella, Dr Carla Obradors, Dr Guanqi Qiu and Dr Peter Štacko – setting the stage for the second poster session. The posters offered again a lively snapshot of the remarkable scientific breadth of the conference: from emerging carbyne and carbene reactivity to photoredox, electrochemical and biocatalytic strategies; from protein editing and engineered enzymes to therapeutic nanomaterials, molecular switches and MOFs. Conceptual physical organic chemistry also entered. Digital chemistry, industrial catalysis and precious-metal recycling completed the picture, highlighting how modern synthesis increasingly thrives at the boundaries between disciplines. Discussions around the posters remained lively and thought-provoking throughout the session, with many conversations naturally spilling over into the final conference dinner.

## Photoactive materials à la carte: doping aromatics by design

The Wednesday evening session, chaired by Conference President Prof. Melchiorre, saw Prof. Davide Bonifazi (University of Vienna) take to the stage, and deliver a lecture entitled “*Harnessing Photons: Tailoring Aromatic Systems for Light-Driven Chemistry*”. The talk showcased the Bonifazi group’s target-oriented synthesis of heteroatom-doped π-conjugated aromatic systems and illustrated how precise molecular design can be exploited to tailor the photophysical properties of organic materials for light-driven applications.

Prof. Bonifazi first challenged conventional static molecular descriptors – including ground-state geometry, frontier orbital gaps, transition dipoles and oscillator strengths – with the dynamic reality of conformational and thermally induced fluctuations, emphasising how molecular motion can govern excited-state behaviour and reactivity. A central theme of the lecture was the use of heteroatom substitution, in particular the replacement of C=C motifs with polarised B–N units, to tune the electronic structure and photophysics of aromatic scaffolds.^63^ These concepts were illustrated through recent work on BN-doped peri-acenoacene ribbons, where elongation of the oligoarylenyl backbone produces bathochromically shifted absorption and emission and gives rise to thermally activated delayed fluorescence, and on BN/BO-doped peri-acenoacene congeners, in which peripheral heteroatom doping modulates π-conjugation, excited-state structure and emission properties.^64,65^ Finally, Prof. Bonifazi highlighted how structural distortion and conformational dynamics can be exploited as design elements for distortion-induced TADF and conformational gating of emission.

Prof. Bonifazi illustrated that aromatic systems are best regarded as dynamic molecular landscapes, in which atomic composition, conformation, and structural distortion act in concert to govern photophysical behaviour. Engineering and mastering these design elements opens new opportunities for the rational development of next-generation functional photoactive materials.

## Take a leaf out of Nature’s book

The final scientific session of the 59th Bürgenstock Conference opened on Thursday morning under the chairmanship of Prof. Massimo Bietti, beginning with a remarkable lecture by Prof. Xiaoguang Lei (Peking University). Entitled “*Opportunities in Merging Chemical and Biological Synthesis*”, the talk showcased the Lei group’s efforts to disclose biosynthetic enzymes, shed light on Nature’s synthetic pathways and engineer biocatalysts capable of streamlining drug manufacturing and reshaping the reactivity and selectivity of conventional organic reactions.

The first part of the lecture focused on the biosynthesis of complex natural products with pharmaceutical applications. Prof. Lei’s work challenged the remarkable efficiency with which Nature assembles the taxol scaffold from geranylgeranyl pyrophosphate (GGPP), requiring only nine enzymes to produce baccatin III – a direct precursor to the taxol framework. The Lei group identified previously missing enzymes in the biosynthetic pathway, including a bifunctional cytochrome P450 responsible for an unprecedented oxidative rearrangement involved in the formation of taxol’s oxetane moiety, and an enzyme mediating its C9 oxidation.^66^ Building on these discoveries, the group re-assembled the baccatin III biosynthetic pathway in *Nicotiana benthamiana*, an herbaceous plant of the tobacco family, by injecting the starting materials and the nutrients directly into its leaves – thereby effectively turning leaves into living reactors for natural-product synthesis. A second case study centred on the production of homoharringtonine, a clinically used treatment for leukaemia derived from the alkaloid (−)-cephalotaxine. By identifying missing biosynthetic enzymes, the Lei group reconstructed the enzymatic route towards cephalotaxinone and homoerythratine and uncovered two highly homologous cytochrome P450 enzymes capable of divergent oxidation pathways.^67^ Pinpointing the key residues responsible for their contrasting selectivity ultimately enabled the complete biosynthetic pathways to both alkaloids to be reconstituted in *Nicotiana benthamiana* leaves.

Prof. Lei then turned from biosynthesis to enzyme engineering, targeting what he described as “*the most important functional group in drug discovery*”: the amide bond. Inspired by aldehyde dehydrogenases, the group repurposed these enzymes into oxidative amidases capable of directly coupling aldehydes and amines through oxidative amidation. Five rounds of directed evolution reshaped the enzyme’s active site to accommodate amine nucleophiles, while coupling the evolved catalyst with an alcohol dehydrogenase enabled a two-enzyme cascade that converts readily available primary alcohols directly into amides.^68^ The remarkable chemoselectivity and efficiency of this platform enabled the application of this cascade to the synthesis of several drug molecules. Unpublished studies pushed this concept further, using directed evolution to develop enzymes for enantioselective amidation through the tandem coupling and kinetic resolution of amines bearing racemic α-stereocentres. Remarkably, the engineered enzymes could overturn conventional solution-phase selectivity: for substrates bearing both primary and secondary amines, amidation occurred selectively at the less nucleophilic secondary amine.

Prof. Lei’s lecture demonstrated that, rather than merely learning from Nature, chemists can actively team up with plants and enzymes to devise more efficient and selective routes to complex bioactive molecules and pharmaceuticals. From tobacco leaves as living reactors to engineered enzymes that rewrite textbook selectivity, the talk offered a glimpse of a future in which chemical and biological synthesis increasingly operate as a single synthetic toolbox. This perhaps brings chemistry back to one of its greenest and most primordial laboratories: the leaf!

## Boron gets radical

*Dulcis in fundo*, the conference concluded its scientific programme with a lecture by Prof. Daniele Leonori (RWTH Aachen) entitled “*Novel Synthetic Methods Using Photochemistry and Photocatalysis*”. The lecture highlighted the Leonori group’s pioneering efforts to harness the underexplored radical reactivity at boron to develop new boron-based reagents that overcome long-standing stability and selectivity challenges in (hetero)aromatic functionalisation, while uncovering new reactivity manifolds for boron-centred radicals that directly convert ubiquitous amine derivatives into reactive carbon-centred radicals through deaminative activation.

The first part of the lecture focused on the unique reactivity of amine-ligated boryl radicals as an orthogonal platform for the borylation of (hetero)arenes.^69^ Unlike conventional transition metal-catalysed C–H borylation, these radical processes provide complementary site-selectivity, enabling the efficient functionalisation of medicinally relevant aza-arenes, aromatics, alkenes and imine scaffolds.^70^ In particular, Prof. Leonori demonstrated that the resulting azine–borane adducts provide an elegant solution to the long-standing “2-pyridyl problem” in Suzuki–Miyaura cross-coupling, owing to their exceptional resistance towards protodeboronation, while serving as versatile boron nucleophiles in Pd-, Cu- and Ni-catalysed cross-coupling reactions.^69,70^

The lecture then turned to the use of amine–borane complexes as versatile radical precursors for deaminative metal-catalysed cross-couplings. Here, amine-ligated boryl radicals, generated *via* HAT from amine–borane complexes, undergo β-scission to cleave the adjacent C–N bond and generate alkyl radicals that engage in copper-catalysed cross-coupling reactions with a broad range of nucleophilic partners, effectively converting native amines into a variety of valuable functional groups.^71^ Prof. Leonori further illustrated the versatility of this platform through its extension to the enantioselective deaminative cyanation of benzylamines, and to the deconstructive functionalisation of unstrained saturated cyclic amines, providing streamlined access to remotely functionalised linear amines from ubiquitous pyrrolidine and piperidine scaffolds.

Prof. Leonori’s findings demonstrate that boron chemistry extends far beyond its established role in homologation and cross-coupling reactions. By rediscovering and harnessing the unique reactivity of boron-centred radicals, Leonori and his team have expanded the synthetic toolbox for aromatic functionalisation and amine editing, revealing boron to be not only an exceptional coupling partner but also a powerful platform for the design and discovery of radical reactions.

## Final remarks

The 59th Bürgenstock Conference concluded with closing remarks from this year’s President, Prof. Paolo Melchiorre, who thanked the attendees, speakers, organising committee and, in particular, the four student helpers – Anastassia, Evelyn, Josh and Nicolas – whose enthusiasm, proactivity and dedication throughout the week ensured the smooth running of both the scientific discussions following each lecture, and the conference as a whole. Reflecting on this edition, Prof. Melchiorre celebrated the exceptional scientific breadth, intellectual intensity and collegial atmosphere that had characterised the five days, expressing his satisfaction that the spirit which has made the Bürgenstock Conference unique for six decades had once again been preserved.

The closing session also looked ahead to the future of the conference. Prof. Christof Sparr was announced as the new JSP Fellowship coordinator, succeeding Prof. Jérôme Waser, while Prof. Varinder Aggarwal was introduced as President of the 60th Bürgenstock Conference. Inviting everyone to reconvene next year, Prof. Aggarwal promised “*five superb and inspiring days of chemistry*” and, reassuringly, “*a party every night*”. Supporting him as Vice President will be Prof. Jérôme Lacour. These appointments were warmly welcomed by the audience and reaffirmed the Bürgenstock community’s enduring commitment to scientific excellence, international collaboration and support for the next generation of researchers.

As participants departed Brunnen, they took home far more than notebooks filled with chemistry scribbles: they left with fresh ideas, new collaborations, renewed inspiration and the anticipation of meeting again next year to celebrate the 60th edition of one of chemistry’s most distinctive conferences.
